# Structural Analysis of Technical-Tactical Elements in Table Tennis and their Role in Different Playing Zones

**DOI:** 10.1515/hukin-2015-0076

**Published:** 2015-10-14

**Authors:** Goran Munivrana, Lidija Zekan Petrinović, Miran Kondrič

**Affiliations:** 1 University of Split, Faculty of Kinesiology, Croatia.; 2 University of Zagreb, Faculty of Kinesiology, Croatia.; 3 University of Ljubljana, Faculty of Sport, Slovenia.; 4 International table tennis federation, Lusanne, Suisse

**Keywords:** racquet sports, motor skills, expert analysis, Kruskal-Wallis test

## Abstract

For the purpose of determining the overall structure of technical-tactical elements in table tennis and evaluating their role in different playing zones around the table, a new measuring instrument (a questionnaire) was formulated that took advantage of the expert knowledge of top, world class table tennis coaches. The results of the hierarchical taxonomic (cluster) analysis showed that the overall structure of the technical-tactical elements forming the table tennis technique could be divided into three basic groups; a group of technical-tactical elements (A) used in the phase of preparing one’s own and disabling the opponent’s attack; a group of technical-tactical elements (B) used in the phase of attack and counterattack; and a group of technical-tactical elements (C) used in the phase of defense. The differences among the obtained groups of table tennis elements were determined by applying the Kruskal-Wallis test, while relations between the groups and their role in different playing zones around the table were analyzed by comparing the average values of the experts’ scores.

## Introduction

Table tennis is considered to be one of the most demanding sports games when viewed in terms of its structural complexity in comparison with other sports disciplines. It is extremely complex taking into consideration technical and tactical aspects as it demands a wide range of technically different strokes which, among other things, depend on the material (type of rubber) with which a stroke is made, and the type of stroke made by the opponent. Therefore, studies of players’ technical-tactical activities assume a key role in the structural analysis of table tennis.

While team sports games have attracted a relatively large number of research studies determining and analyzing the role of various technical-tactical structures and elements in a game, there are much fewer studies concerning racquet sports (O’Donoghue, 2001; Lees, 2002, 2003; Cabello-Marinique and Gonzales-Badillo, 2003; Zhang and Hohmann, 2004; Zhang et al., 2007; Yu et al., 2010), including table tennis. In previous research studies conducted in table tennis on the structural characteristics of the game, technical-tactical actions during competition had been evaluated (Méndez Patiño et al., 2010; Pfeifer et al., 2010; Pradas et al., 2010; Zhe et al., 2010), different types of players’ technical-tactical activities in matches had been analyzed (Galina, 1992; Guan et al., 2011; Djokić, 2001, 2007; Dong, 2007; Hao et al., 2007; Zhe et al., 2007; Yu et al., 2008; Wang et al., 2009; Poizat et al., 2012), and the role of certain technical-tactical elements and the characteristics of certain playing styles had been evaluated (Drianovski and Otcheva, 2000; Sun, 2007; Zhao X. et al., 2007; Zhao H. et al., 2007 etc.). The data had primarily been collected by means of video analyses of table tennis matches.

Unlike all of the above-mentioned table tennis research studies which analyzed and evaluated the role of only a limited number of technical-tactical contents or activities in the game, the aim of this study was to determine the hierarchical structure of the overall group of technical-tactical elements used in table tennis and to evaluate their role (frequency of play and effectiveness) in different playing zones on and around the table.

When seeking to establish and scientifically analyze the hierarchical structure of the overall group of technical-tactical elements used in table tennis, one of the main issues is choosing the most appropriate method for collecting the data. The main “problem” in collecting data in a table tennis game (like in all sports games) is that there are always two opponents (or teams) confronting each other and, therefore, the data obtained from the matches also depend directly on the quality of the opponent (Hudetz, 2003). For that reason, it is very difficult to obtain from a video analysis of table tennis matches an objective image of the real value of all technical-tactical elements used in a table tennis game (even if a large sample of matches is observed) as the data obtained merely represent a partial or relative value in the observed matches.

Since a single match only generates a limited amount of information, in order to determine the overall group and more reliably evaluate the importance and role of each individual technical-tactical element, one should statistically analyze a huge sample of matches, point by point, and note every technique performed. In doing so, one should also ensure that players with different styles (systems) of play meet and play with different materials (rubbers) so as to enable all table tennis techniques to appear in the sample in order for them to be adequately evaluated. It is evident that such an approach would present the researcher with huge organizational problems when collecting data in terms of the vast use of time and means, whilst even then it is still uncertain that it would be possible to fully cover the entire group of technical-tactical knowledge and include all the factors that determine the real value and role of the technical-tactical elements of table tennis.

Having in mind the aims and extent of this research, the authors chose a new approach to solve the mentioned data collecting problem. A new method (compared to those used in previous research studies conducted in table tennis) was applied in this research that took advantage of the expert knowledge of top table tennis coaches in order to establish the overall structure of the technical-tactical elements used in table tennis, and to evaluate their importance and role in different playing zones around the table.

For this purpose, a measuring instrument (a questionnaire) was formulated in order to gather a large pool of empirical expert knowledge (which the experts had acquired through decades of top-level involvement in the sport) which should enable the collection of the largest quantity of information needed to achieve the aims of the research.

## Material and Methods

### Sample of entities

The sample of entities comprised technical-tactical elements in a table tennis game that had been selected by the authors on the basis of information in the professional literature related to the systematization, i.e. division of technical-tactical elements (Harangozo, 1963; Hudetz, 2000, 2003; Wohlgefahrt, 2004; Molodzoff, 2008; Zhan et al., 2012), before the sample was presented to table tennis experts who amended and approved the selection.

All of the elements in the sample were initially derived from 8 basic table tennis techniques (“Drive” attack; Topspin attack, Block, Backspin defense, Chop, Attack over the table “Flick”, Balloon defense, Service) and were meant to cover all possible technical and tactical applications of each of the basic techniques.

In table tennis, all strokes (apart from service) are performed at a ball coming from the opponent’s side. Therefore, the systematization of the technical-tactical elements depended on the type of ball a certain stroke is performed at. Opponent’s balls vary in their speed, rotation, flight path, and landing location (placement) so they also require a player to use different techniques for the same basic stroke. As a result, despite belonging to the same basic family, some strokes represent separate techniques since there are significant differences among them in both performance techniques and the tactical effects sought. Based on these criteria, 110 technical-tactical elements (listed in the appendix) best representing the entire group of motor knowledge in table tennis were selected, with the aim to cover all possible technical and tactical applications of each of the 8 basic techniques.

### Sample of variables

The sample of variables includes 18 variables divided into six basic groups they were derived from, with each representing an individual segment of a table tennis game (*1. Systems of play; 2. Playing zones (spaces) around the table; 3. Game phases; 4. Racquet grip styles; 5. Materials used in the game; and 6. Basic tactical means*). The selected variables seek to describe the basic characteristics (attributes) of a table tennis game with which it is possible to significantly distinguish the technical-tactical elements.

#### 1. Systems of play (basic)

➢ Attack in the table zone (BSPATZ) – an offensive playing system mainly characterized by short and fast attacking techniques carried out from a distance next to the table (up to a maximum of 1 m from the table).
➢ Attack from a half distance (BSPAHD) – an offensive playing system above all characterized by attacking techniques executed at middle distances (1–2 m from the table).
➢ Defense (BSPDEF) – a defensive playing system largely characterized by defensive techniques executed at greater distances (more than 2 m from the table).

Variables within this group encompass three basic playing systems which best combine the various playing concepts used in modern table tennis. Although the professional literature (Hudetz, 2003; Wohlgefahrt, 2004; Molodzoff, 2008) outlines various systematizations and classifications of systems of play, for the purpose of this study such divisions were consolidated into three basic playing concepts (within which there were different variations) which all of the experts had agreed exist in modern table tennis (in varying proportions in table tennis for men and women). The purpose of this group of variables was to establish the importance of a single technical-tactical element for a certain system of play.

#### 2. Playing zones (spaces around the table)

➢ Zone “A” outside the table (PZZON «A») – the zone next to the table, up to a maximum distance of 1 m from the table.
➢ Zone “B” outside the table (PZZON «B») – the zone of half distance where strokes are played from a distance of around 1–2.5 m from the table.
➢ Zone “C” outside the table (PZZON «C») – the zone of distance where strokes are played at distances exceeding 2.5 m from the table.

Variables of this group reveal three playing zones divided according to the positions in which certain techniques are used in relation to the table surface. This division reflects the information available in the professional literature (Hudetz, 2003; Wohlgefahrt, 2004; Molodzoff, 2008), as well as the interviews with the experts. The aim of these variables is to ascertain how successfully a single technical-tactical element is performed from different zones around the table.

#### 3. Game phases

➢ Offensive phase (GPHOFF)
– Attack with offensive strokes at defensive balls
➢ Passive defense phase (GPHDEF)
– Defense with defensive strokes at offensive balls
➢ Active defense phase – counterattack (GPHCAT)
– Counterattack with offensive strokes at offensive balls
➢ Phase of preparing one’s own and disabling the opponent’s attack (GPHPRD)
– Performing techniques which do not have a distinctly pronounced defensive or offensive component, but are used to disable a successful attack by the opponent or prepare a favorable situation to execute his/her own attack

The variables in this group encompass four basic technical-tactical phases in performance of the game and aim to establish how successfully a certain technical-tactical element is employed in a particular game phase.

#### 4. Racquet Grip styles

➢ Shake hand grip/classical racquet grip (RGSCLA)
➢ Penholder grip (RGSPEN)

The two variables in this group describe two basic ways of holding a table tennis racquet. Their aim is to establish how much each racquet holding technique affects the performance of a certain technical-tactical element.

#### 5. Materials (racquet rubbers) used in the game

➢ Pimples-in rubber and sponge – “backside” (MATBAC): an inverted rubber with pimples made from the most versatile rubber type that is able to generate tremendous spin due to its smooth and tacky surface. It is especially suitable for all styles of play from the all-out attacker to the most defensively minded chopper.
➢ Short pimples-out rubber and sponge – “soft” (MATSOF): an inverted rubber turned upside down with the pimples out that enables a player to take some of the spin off from the opponent’s ball and allows aggressive attacking of the opponent’s shots regardless of the oncoming spin. It is very useful for hitting, blocking and returning serves, but is unable to produce as much spin as an inverted (“backside”) rubber.
➢ Long pimples-out rubber – “grass” (MATGRA): an inverted rubber turned upside down with the pimples out, very similar in composition to short pip rubbers, although the pips are taller with the chief characteristic of reversing the oncoming spin. It is generally used by defensive players who rely on their opponents to make mistakes.

The variables in this group describe three basic types of rubber with different characteristics used in table tennis of which aim is to determine how successfully a certain table tennis technique can be performed with a particular type of rubber.

#### 6. Basic tactical means

➢ Ball speed (BTMSPE)
➢ Ball placement (BTMPLA)
➢ Ball rotation (BTMROT)

Variables in this group describe three basic tactical means players have available when realizing their own tactical ideas. They aim to establish the extent of the role of a single tactical means in the performance of an individual technical-tactical element.

### Selection of the experts

The selection of coaches/experts was carried out according to very strict result criteria, with a condition that a trainer considered an expert had been a leading male or female player or member of a national team that had won a medal at the largest international table tennis competitions (European Championships, World Championships, Olympic Games, European TOP 12), or whose club team had played in the finals of a European club competition (European Champions League, ETTU Cup, Europe Super Cup). In line with these criteria, eight top table tennis trainers (experts) were selected and they agreed to participate in this research.

### Measuring instrument

In order to collect the data for the purpose of this research where experts evaluated the importance and role of technical-tactical elements in table tennis, a measuring instrument (a questionnaire) used for researching personal opinions was formulated. The questionnaire is based on the measuring technique of scaling, where the scale is made up of five numerically and descriptively expressed categories (a Likert scale from 1 to 5). They are classified so that each represents a certain level, i.e. they differ from the previous one by intensity, starting from the lowest to the highest degree.

An example of the measuring scale:

How successfully (frequently and effectively) is a single technical-tactical element played from a certain playing zone on and around the table?

1. Not played at all or played extremely rarely
2. Occasionally played (below-average playing frequency and effect)
3. Average playing frequency and effect
4. Very frequently and effectively played (above-average success rate)
5. Exceptionally frequently and effectively played (high above-average success rate)

The table tennis experts were asked to give their opinions in the form of numerical answers in the questionnaire. By circling one of the scores, they evaluated the importance of every single technical-tactical element from the sample of entities (listed in the appendix) in relation to every single variable and, thus, 1,980 scores per expert were recorded (the rating values of the 110 technical-tactical elements in relation to the 18 variables).

### Data-processing methods

After the data were collected (average values of the experts’ scores recorded for an element on each of the 21 variables describing the six basic segments of a table tennis game), all of the experts’ scores (1,980 scores per expert, 15,840 scores in total) were entered into the matrix before the following data-processing methods were applied:

1. Determining the metrical characteristics of the variables
  This entailed determining the level of agreement among the experts (test particles) in the evaluation of common metrical characteristics (objectivity and homogeneity) in both classical and Guttman’s models (of measuring), as well as determining the sensitivity (discriminative ability) of the measuring instrument by analyzing the basic descriptive (M, SD, Mdn, MIN, MAX) and distributional (K-S, MaxD) statistical parameters of the variables after condensing the individual experts’ scores (test particles) into one unique common score.
2. Analysis of the grouping technical-tactical elements
  This involved a hierarchical classification of technical-tactical elements into homogeneous groups by using taxonomic (cluster) analysis, the Ward’s method or the minimum variance method (Ward, 1963), which amounted to calculating the minimum sum of square discrepancies of any of the two hypothetical entity groups. This approach performed better than other methods for hierarchically grouping objects (Jain and Dubes, 1988).
3. Determining the differences and relations among the obtained groups of table tennis elements
  Here differences were determined among the obtained groups of technical-tactical elements (results of the taxonomic analysis) in variables describing the *playing zones around the table* by applying the *Kruskal-Wallis test*, a non-parametric method that is a nonparametric equivalent of a one-way analysis of variance but, unlike ANOVA, does not assume a normal distribution of the residuals. The relations among the obtained groups were determined with an analysis of their implicated relations by comparing the average values of their scores.

## Results

### Determining the experts’ level of agreement in evaluations of the common subject of measurement

Table 1 shows the level of agreement among the experts (test particles). Their objectivity was determined by analyzing several different reliability coefficients, using the classical and Guttman’s measuring method, as well as the representation and homogeneity of the experts when determining the common subject of measurement.

The results show that the experts had a high level of agreement, with the variable describing the classical racquet grip (RGSCLA) being the only exception. For all the other variables, the experts revealed admirable objectivity (the measurement reliability level exceeded 0.90) and homogeneity in determining the common subject of measurement independently from the applied measuring model (classical or Guttman’s).

### Analysis of the descriptive and distributional parameters of the variables

Table 2 presents the basic descriptive and distributional statistical parameters of all variables after condensing the results of their particles into a unique common measuring result with the Burt’s simple summation method (Burt, 1941 as cited in Momirović et al., 1999).

The variables describing playing zones around the table were not evenly distributed across the measurement scale. Therefore, the distribution of the results for those variables differed significantly from values characterizing the normal distribution of the results.

### Analysis of grouping the technical-tactical elements

**Figure 1:**
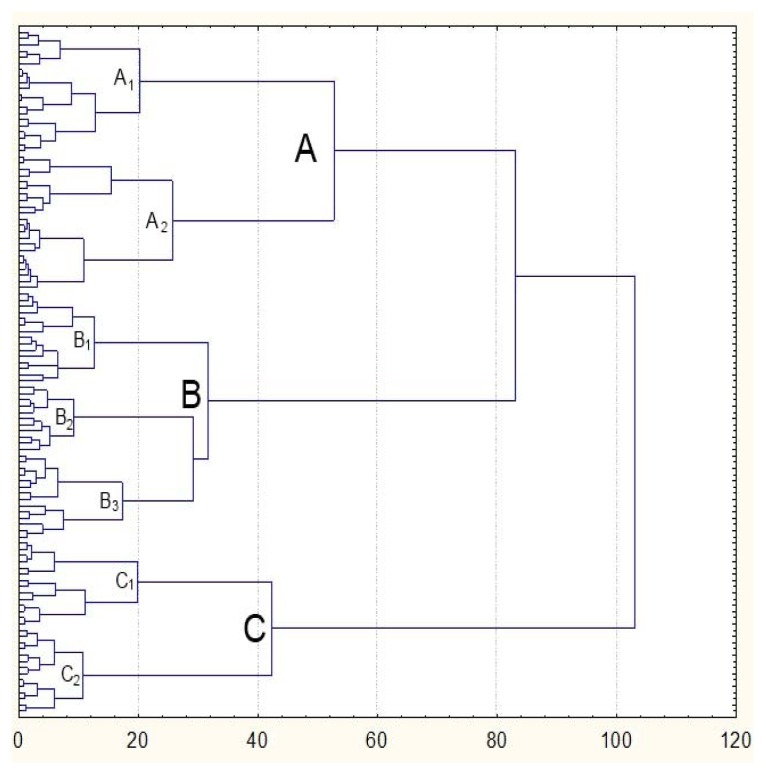
Hierarchical structure of technical-tactical elements in a table tennis game after grouping them in clusters using the Ward’s method

Figure 1 presents the structure of the grouped technical-tactical elements by applying a hierarchical taxonomic analysis (Ward’s method) in the area of selected variables describing a table tennis game. The grouping of the technical-tactical elements was based on the resemblance of their individual profiles (rows of the data matrix) over the whole set of variables describing various aspects of a table tennis game.

The technical-tactical elements were classified in three basic groups (A, B and C) according to similarities in the technical-tactical characteristics.

- Group (A) contained technical-tactical elements used in the phase of preparing one’s own and disabling the opponent’s attack
- Group (B) included technical-tactical elements used in the phase of attack and counterattack
- Group (C) encompassed technical-tactical elements used in the phase of defense

Within the basic groups (A, B and C), the technical-tactical elements could be further divided into sub-groups (A1, A2; B1, B2, B3; C1, C2), which had even more homogeneous common characteristics (Table 3).

### Determining the differences between the obtained groups of technical-tactical elements and the evaluation of their role in different playing zones around the table

Since the distribution of the results for all variables describing playing zones around the table differed significantly from values characterizing the normal distribution of the results (Table 2), the differences among the groups obtained by means of taxonomic analysis (Figure 1, Table 3) were determined by applying the Kruskal-Wallis test.

**Figure 2:**
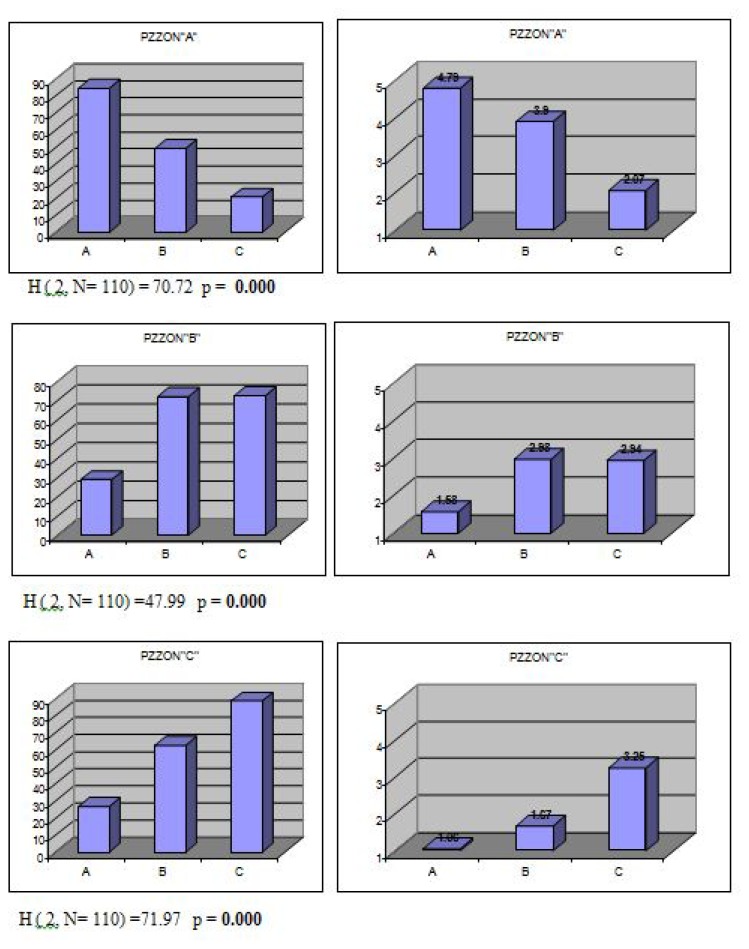
Diagrams of the average mean ranks (on the left) and mean values of the experts’ scores (on the right) of the three basic groups of technical-tactical elements in the variables describing playing zones around the table (PZZON”A”, PZZON”B” and PZZON”C”)

**Figure 3:**
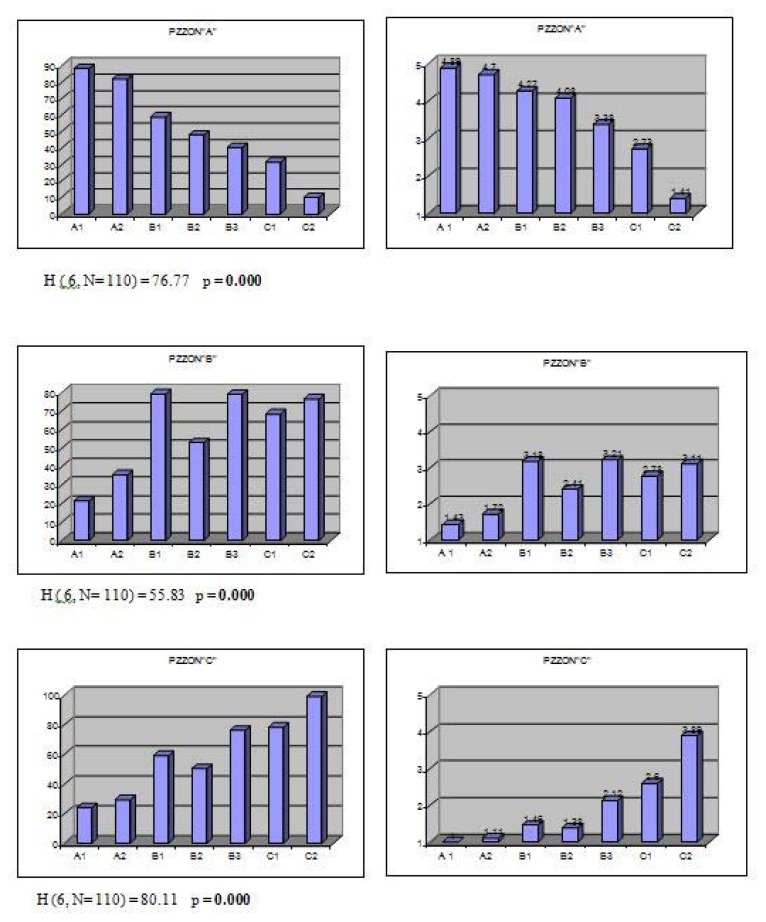
Diagrams of the average mean ranks (on the left) and mean values of the experts’ scores (on the right) of the seven subgroups of technical-tactical elements in the variables describing playing zones around the table (PZZON”A”, PZZON”B” and PZZON”C”)

Figures 2 & 3 present the differences (results of the Kruskal-Wallis test) among the obtained groups and subgroups of technical-tactical elements as well as the importance and role (by comparing the mean values of the experts’ scores) each group had in different playing zones around the table.

The results of the Kruskal-Wallis test show that the obtained groups and subgroups of technical-tactical elements differed significantly in all variables describing playing zones around the table.

One can see from the arithmetic means of the experts’ scores in the obtained groups and subgroups of technical-tactical elements the importance and role each group had in different playing zones around the table.

## Discussion

The aim of this research was to establish the structure of technical-tactical elements in table tennis, and evaluate their importance and role in different playing zones on and around the table. The data were obtained by collecting and analyzing the expert knowledge of selected top table tennis coaches, with their expertise being ensured by very strict criteria for their selection.

The experts strongly agree (Table 2) on evaluating the common subject of measurement (the importance and role of the technical-tactical elements in table tennis) as for all variables, except for the variable describing the classical racquet grip (RGCLAS), they demonstrate very high objectivity (the level of measurement reliability exceeds 0.90) and homogeneity, independently of the measurement model applied (classical or Guttman’s).

In the variable describing the traditional racquet grip (RGCLAS), a low level of reliability is achieved on those coefficients based on the classical measurement theory, while in the Guttman’s model the reliability is very good even for this variable. Due to its diversity, the classical racquet grip facilitates the successful performance of all table tennis techniques and facilitates playing in all systems of play (which, for instance, is not the case with the penholder grip). Therefore, all results for this variable are situated in the upper half of the measurement scale (above a score of 3) as all technical-tactical elements in table tennis can be performed well using this most popular racquet grip technique. The weak variability of the results negatively influences the sensitivity of the measurement instrument, and thereby also the homogeneity of the experts who, within such a narrow range of results, define the main subject of measurement differently so that the overall variance breaks down. As a result, the variable RGCLAS is the only variable for which the results of the experts’ scores are not situated on the same main component (Table 2).

The outcome of the hierarchical taxonomic analysis (Figure 1) reveals that the total structure of technical-tactical elements in table tennis can be divided into three basic homogeneous groups of elements.

The first group (group A) consists of 42 technical-tactical elements used in preparing one’s own and disabling the opponent’s attack. The list of technical-tactical elements making up this biggest group includes those technique elements that represent the basis of the first playing phase, i.e. technical-tactical elements such as placing the ball in the game (service), returning the service ball (return), as well as elements with which, during a point, one attempts to prepare one’s own attack and simultaneously disable a successful attack by an opponent (“short at short”, “chop”, “flick”). The results of Djokic’s studies (2001, 2007) conducted on a sample of 70 top table tennis players in 35 matches at leading World and European competitions show that the effective realisation of service advantage and successful return of an opponent’s service are the key factors that influence a player’s success in the modern, top-level table tennis game. The technical-tactical elements in this group (group A) can also be divided into two basic sub-groups with more homogeneous common characteristics which are, in relation to the place from where they are played, divided as follows:

- a group of technical-tactical elements (A1) used in the preparation of one’s own and disabling the opponent’s attack, and played above the surface of the table (“flick”, “short at short”, offensive chop); and
- a group of technical-tactical elements (A2) used in the preparation of one’s own and for disabling the opponent’s attack, and played outside the surface of the table (service, defensive chop).

The second group (group B) comprises 40 technical-tactical elements used in the phase of attack and counterattack as very offensive techniques. The list of technical-tactical elements in this group includes offensive techniques used or with the aim of direct scoring (final topspin or “drive” attack strokes – smashing), gaining or keeping the advantage (initial topspin, topspin at topspin counterattack, drive counterattack or active block), i.e. gameplay initiative (continuation of topspin or strong “drive” attacks). According to Pfeiffer et al. (2010) offensive and counteroffensive phases of the game are the two most important game phases in table tennis. Consequently, attacking and counterattacking techniques are the most effective techniques for winning the point in a table tennis game. Technical-tactical elements in this group (group B) can be divided into three basic subgroups with even more homogeneous common characteristics:

- a group of offensive technical-tactical elements (B1) played in the attack phase at the opponent’s defensive balls, and characterized by playing strokes with a great forward rotation of the ball (attack with rotation – topspin);
- a group of offensive technical-tactical elements (B2) played in the attack phase at the opponent’s defensive balls, and characterized by playing strokes without a ball rotation (attack without rotation – drive attack); and
- a group of offensive technical-tactical elements (B3) played in the counterattack phase at the opponent’s offensive balls (active block, drive counterattacks, topspin at topspin).

The third group (group C) contains 28 technical-tactical elements employed in the phase of defense as defensive techniques. The list of technical-tactical elements in this group includes defensive techniques used as basic techniques in the defensive system of play (backspin defense), or as “rescue” techniques in difficult situations when the opponent has the initiative in a point (passive block, flat balls, balloon defense). The technical-tactical elements in this group (group C) can be divided into two basic sub-groups with more homogeneous common characteristics:

- a group of defensive technical-tactical elements (C1) played in the phase of defense in situations of an opponent’s evident initiative, chiefly with the aim of returning the ball into the game at any cost and thereby remaining in the point (passive block, flat balls, balloon defense); and
- a group of defensive technical-tactical elements (C2) mainly used as basic techniques in the defense system of play (although they are sometimes also used as “rescue” techniques in offensive systems), as a playing style whereby the player goes tactically and consciously into defense and defends him/herself from the opponent’s offensive balls (backspin defense).

The distribution of the results for variables describing playing zones around the table differs significantly from values characterizing the normal distribution of the results and so the data are unsuitable for a parametric test and differences among the obtained groups were determined by applying the Kruskal-Wallis test. The Kruskal-Wallis test results show that the groups of technical-tactical elements obtained with the hierarchical taxonomic analysis (at the level of the three basic groups (Figure 2) differ significantly for all variables describing playing zones around the table.

At the level of the seven sub-groups (Figure 3), the differences among the obtained groups are significant so the obtained groups may be considered as groups of elements with different technical-tactical characteristics in relation to each of the variables describing playing zones around the table.

The analysis of the average (mean) values of scores helps determine the importance and role of the obtained groups of technical-tactical elements in playing zones around the table.

The results (Figure 2) show that elements of the preparation of one’s own and disabling the opponent’s attack (group A) can only be effectively played from the zone next to the table (PZZON “A”). All of the technical-tactical elements that form group A (A1 and A2) are played extremely effectively (average score 4.79) from the zone next to the table (PZZON “A”) as the techniques of service, “flick”, “short at short ball”, offensive and defensive chop can be efficiently performed only from this zone, up to a maximum distance of 1 m from the table. At distances greater than 1 m from the table, elements from group A cannot be used effectively (PZZON“B”) or cannot be used at all (PZZON “C”).

The second group of technical-tactical elements, elements of attack and counterattack (group B), are the most effective if executed from the zone next to the table (PZZON“A”). In this zone, up to a maximum distance of 1 m from the table elements of attack and counterattack are played extremely (groups B1&B2) or at least very effectively (group B3) (Figure 3). Offensive attacking strokes/techniques are the most powerful when executed from the zone next to the table, and at the same time an opponent has the least amount of time to react to a fast oncoming ball and effectively play his own stroke.

With an increase in a player’s distance from the table, offensive attacking strokes/techniques gradually lose some of their power and precision and become less effective. Although some of the stroke power is lost when a player moves away from the table, elements of attack with rotation – topspin (group B1) and elements of counterattack (group B3) are still performed very effectively (Figure 3) from the zone of half distance (PZZON“B”). In the zone of half distance a player is still close enough to the table to perform attacking topspin techniques and all counterattacking techniques with substantial power and precision and has more time available to react to the opponent’s balls than from the zone next to the table (PZZON“A”). The zone of half distance (PZZON“B”) is an ideal playing zone for players using both side topspin attacks as the dominant playing style. Nowadays, topspin offensive and counteroffensive game actions are the main winning strategies in top table tennis for men, as noted by Pfeiffer et al. (2010).

On the other hand, elements of drive attack without rotation (group B2) are played much less effectively (below-average effectiveness; average score 2,41) from the zone of half distance (PZZON“B”), above all smashes in response to suitably higher balls. Since the angle between the highest point of the ball bounce and the table surface becomes smaller, it becomes much more difficult to strongly and precisely perform drive attacks from a half distance and obviously even more so from the zone of distance (PZZON”C”).

From the zone of distance (PZZON”C”), of all the offensive attacking technical-tactical elements that form group “B”, only the counterattacking techniques (group B3) are played with substantial frequency and effect (Figure 3). Unlike the other attacking techniques, the counterattacking techniques are the only ones (group “B”) played at opponents’ fast offensive balls and that is why a player (when not in a perfect position to perform a counterattack in the zones closer to the table) often needs to have greater distance between himself and an opponent to have more time to react to a fast approaching ball and perform a successful counterattack.

Elements played in the phase of defense (group C) are used in all three playing zones outside the table but, unlike the offensive attacking techniques, their frequency of play and effectiveness grow with an increase in a player’s distance from the table (Figures 2 & 3). This is logical since a player, when ending up in the phase of defense (consciously or forced to by an initiative of the opponent), wants to be at some distance from the table to have more time to react to the opponent’s fast approaching offensive balls.

On the level of the subgroups, it is evident that the techniques of passive defense (group C1), which are primarily used as “rescue” and “survival” techniques in those situations where a player is forced to do so due to an initiative of the opponent, are almost equally used in all three basic playing zones outside of the table. The passive block techniques are usually played in zones closer to the table (Figure 3), while the balloon defense and flat balls techniques are played from the zones of half distance and distance from the table.

In contrast, the techniques of backspin defense (group C2) are primarily used as basic techniques in a defensive system of play and are almost exclusively played from zones distant from the table (PZZON”B” and PZZON“C”). In the zone next to the table (PZZON“A”), a player is usually too close to the table to be able to successfully react to the opponent’s fast offensive balls and absorb their power.

The results provided by this research enable a better understanding of the structure of technical-tactical elements in table tennis, as well as an evaluation of their role in specific playing zones (spaces around the table). Since ¬the choice of an appropriate system of play largely depends on player’s abilities (both technical-tactical and anthropological) to more or less successfully perform table tennis techniques in certain zones around the table, the information yielded by this research can be successfully applied in practice, especially when planning long-term technical-tactical training.

## Conclusion

The expert analysis of the structure of the technical-tactical elements in table tennis showed that the whole group of technical-tactical elements forming the table tennis technique can be divided into three basic groups: a group of technical-tactical elements (A) used in the phase of preparation of one’s own and disabling the opponent’s attack; a group of technical-tactical elements (B) used in the phase of attack and counterattack; and a group of technical-tactical elements (C) used in the phase of defense.

Within those basic groups (A, B and C) the technical-tactical elements are divided into subgroups in which they have even more homogeneous common characteristics, which enabled a more precise determination of the role and hierarchical importance of certain groups of table tennis techniques in different playing zones around the table.

Apart from the scientific contribution to technical knowledge of the game, the results of this research also have practical relevance. They provide a variety of information that should be very useful to coaches when choosing the most appropriate playing system for their players, in line with the players’ anthropological and technical-tactical predispositions/abilities, which is extremely important for the successful planning of players’ technical-tactical training.

## Figures and Tables

**Table 1:** Determining the experts’ objectivity and homogeneity when evaluating the common subject of measurement by analyzing several different reliability coefficients with the classical and Guttman’s measuring method

No.	Variable	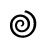 _Cron._	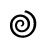 _KC_	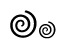	h_1_	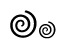 _1_	V_%_	msa
BASIC SYSTEMS OF PLAY								

1.	BSPATZ	0.947	0.948	0.961	1	6.17	77.2	0.994
2.	BSPAHD	0.951	0.952	0.958	1	6.26	78.3	0.995
3.	BSPDEF	0.952	0.954	0.961	1	6.31	78.9	0.995

PLAYING ZONES								

4.	PZZON “A”	0.980	0.981	0.985	1	7.13	89.1	0.999
5.	PZZON “B”	0.941	0.945	0.959	1	6.03	75.4	0.993
6.	PZZON “C”	0.974	0.975	0.982	1	6.99	87.4	0.998

RACQUET GRIP STYLES								

7.	RGSCLA	0.757	0.777	0.963	0	3.38	42.3	0.937
8.	RGSPEN	0.966	0.969	0.974	1	6.84	85.5	0.997

MATERIALS								

9.	MATBAC	0.908	0.912	0.928	1	4.96	62	0.972
10.	MATSOF	0.952	0.953	0.957	1	6.17	77.1	0.994
11.	MATGRA	0.967	0.969	0.976	1	6.78	84.7	0.997

BASIC TACTICAL MEANS								

12.	BTMSPE	0.958	0.960	0.967	1	6.47	80.9	0.997
13.	BTMPLA	0.916	0.919	0.932	1	5.50	68.7	0.986
14.	BTMROT	0.975	0.976	0.985	1	6.96	87.1	0.998

GAME PHASES								

15.	GPHOFF	0.984	0.984	0.989	1	7.30	91.2	0.999
16.	GPHDEF	0.986	0.988	0.991	1	7.48	93.6	0.999
17.	GPHCAT	0.969	0.970	0.983	1	6.78	84.8	0.998
18.	GPHPRD	0.979	0.980	0.985	1	7.17	89.6	0.999

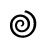
_Cron_ – Cronbach’s coefficient of reliability measured with the classical measuring method on original (Cron.) and standardized (SB) results on the assumption that all particles equally determine the subject of measurement; 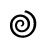
_KC_ – Kaiser-Caffrey’s coefficient of reliability measured with the classical measuring method on standardized values of entities on a linear combination of test particles; 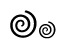
Guttman-Nicewander’s coefficient of reliability measured with the Guttman’s measurement model by transforming the results of entities in particles into universal (Harris’) metrics (Harris, 1962); h_1_ – homogeneity of the test particles based on the number of principal components with positive coefficients of reliability 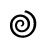
_1_ – first typical value of the correlation matrix among the experts; V_%_ – percentage of common variance of the experts’ opinions; msa – Kaiser-Rice’s coefficient of the experts’ representation determined on the basis of an evaluation of the size of the error expressed as a ratio between the sum of the correlation matrix squares of the anti-image variables and the sum of the correlation matrix squares

**Table 2:** Descriptive statistical parameters for all variables obtained by condensing the experts’ original scores using the Burt’s simple summation method (intact realistic metrics)

No.	Variable	M	Mdn	Min	Max	25%	75%	MaxD	K-S	nd
BASIC SYSTEMS OF PLAY										

1.	BSPATZ	3.10	3.13	1.00	5.00	2.25	4.25	0.101	p< .20	
2.	BSPAHD	3.22	3.38	1.00	5.00	2.63	4.00	0.093	p> .20	
3.	BSPDEF	3.08	3.00	1.00	5.00	2.13	4.00	0.076	p> .20	

PLAYING ZONES										

4.	PZZON“A”	3.69	4.31	1.00	5.00	2.44	4.88	0.208	p< .01	^*^
5.	PZZON“B”	2.51	2.44	1.00	4.50	1.50	3.50	0.159	p< .01	^*^
6.	PZZON“C”	2.00	1.25	1.00	4.88	1.00	2.88	0.232	p< .01	^*^

RACQUET GRIP STYLES										

7.	RGSCLA	4.36	4.38	3.25	5.00	4.13	4.63	0.140	p< .05	^*^
8.	RGSPEN	3.52	4.06	1.00	5.00	2.25	4.63	0.187	p< .01	^*^

MATERIALS										

9.	MATBAC	4.11	4.13	2.75	5.00	3.75	4.63	0.124	p< .10	
10.	MATSOF	3.58	3.69	1.25	5.00	2.88	4.38	0.097	p> .20	
11.	MATGRA	2.12	1.63	1.00	5.00	1.00	3.13	0.227	p< .01	^*^

BASIC TACTICAL MEANS										

12.	BTMSPE	3.21	3.13	1.50	5.00	2.38	4.13	0.105	p< .20	
13.	BTMPLA	3.65	3.81	2.25	4.88	3.00	4.25	0.121	p< .10	
14.	BTMROT	2.84	2.75	1.13	5.00	1.50	4.13	0.147	p< .05	^*^

GAME PHASES										

15.	GPHOFF	2.21	1.38	1.00	5.00	1.13	3.63	0.283	p< .01	^*^
16.	GPHDEF	1.95	1.13	1.00	4.75	1.00	2.63	0.311	p< .01	^*^
17.	GPHCAT	1.85	1.50	1.00	5.00	1.13	1.88	0.264	p< .01	^*^
18.	GPHPRD	2.67	2.19	1.00	5.00	1.25	4.38	0.176	p<.01	^*^

M – arithmetic mean (average value of obtained scores); Mdn – median (middle value of obtained scores); Min – minimum average value of obtained scores; Max – maximum average value of obtained scores; 25% – 75% – interquartile (the range in which there are 50% of central results); MaxD – value of the expected result frequency; K-S – significance of differences between the observed and expected (MaxD) result frequency; nd – ^*^the distribution of results differs significantly from the normal distribution

**Table 3:** Elements classified in certain subgroups after grouping them into clusters using the Ward’s method

Subgroup	Technical-tactical elements
A_1_	1, 95, 3, 2, 96, 4, 61, 62, 92, 91, 63, 64, 93, 94, 67, 68, 101, 102, 103, 104
A_2_	65, 66, 109, 110, 77, 83, 78, 84, 89, 90, 73, 79, 81, 75, 85, 87, 74, 80, 82, 76, 86, 88
B_1_	5, 9, 13, 107, 7, 11, 21, 6, 10, 108, 14, 8, 12, 99, 100
B_2_	15, 97, 17, 105, 19, 16, 98, 18, 20, 106, 22
B_3_	47, 48, 49, 50, 51, 52, 53, 54, 55, 57, 59, 56, 58, 60
C_1_	23, 24, 27, 28, 25, 26, 43, 44, 45, 46, 69, 70, 71, 72
C_2_	29, 30, 33, 34, 37, 38, 41, 42, 31, 32, 39, 40, 35, 36

The numbers for the elements correspond to the elements described in the appendix

## Appendix

**Table:** Sample of selected entities (technical-tactical elements)

Technical-tactical elements
FH attack over the table (“flick”) at a short ball – initial attack
BH attack over the table (“flick”) at a short ball – initial attack
FH attack over the table (“flick”) at a short ball – straight executed flick
BH attack over the table (“flick”) at a short ball – straight executed flick
FH initial topspin at a chop or backspin ball
BH initial topspin at a chop or backspin ball
FH fast final topspin attack (executed with max. force) at a backspin (chop or backspin defense) ball
BH fast final topspin attack (executed with max. force) at a backspin (chop or backspin defense) ball
FH initial topspin attack at a pushed, passive blocked or flat ball
BH initial basic topspin attack at a pushed, passive blocked or flat ball
FH fast final topspin attack at a pushed, passive blocked or lower flat ball
BH fast final topspin attack at a pushed, passive blocked or lower flat ball
FH sidespin (at any defensive ball)
BH sidespin (at any defensive ball)
FH preparatory “drive” attack (without rotation) at a backspin ball (chop or backspin defense)
BH preparatory “drive” attack (without rotation) at a backspin ball (chop or backspin defense)
FH final “drive” attack (without rotation) at a backspin ball (chop or backspin defense)
BH final “drive” attack (without rotation) at a backspin ball (chop or backspin defense)
FH strong final “drive” attack without rotation at a pushed, passive blocked or lower flat ball
BH strong final “drive” attack without rotation at a pushed, passive blocked or lower flat ball
FH final “drive” attack on a higher flat or topspin ball
BH final “drive” attack on a higher flat or topspin ball
FH passive block at an initial topspin or sidespin attack
BH passive block at an initial topspin or sidespin attack
FH passive block at a fast final topspin or strong smash
BH passive block at a fast final topspin or strong smash
FH passive block (at some offensive strokes)
BH passive block (at some offensive strokes)
FH backspin defense on an initial topspin or sidespin attack
BH backspin defense on an initial topspin or sidespin attack
FH pushed defense at an initial topspin or sidespin attack
BH pushed defense at an initial topspin or sidespin attack
FH backspin defense at a fast final topspin or a strong smash (attack without rotation)
BH backspin defense at a fast final topspin or a strong smash (attack without rotation)
FH pushed defense (with min. backspin) at a fast final topspin or a strong smash (attack without rotation)
BH pushed defense (with min. backspin) at a fast final topspin or a strong smash (attack without rotation)
FH backspin defense on an easy drive ball (preparatory attack)
BH backspin defense on an easy drive ball (preparatory attack)
BH pushed defense (with min. backspin) on an easy drive ball (preparatory attack)
FH side backspin defense
BH side backspin defense
FH balloon defense (without rotation) at a final smash or a fast final topspin
BH balloon defense (without rotation) at a final smash or a fast final topspin
FH balloon defense with rotation (topspin or sidespin) at a smash (attack without rotation) or a fast final (executed with max. force) topspin
BH balloon defense with rotation (topspin or sidespin) at a smash (attack without rotation) or a fast final (executed with max. force) topspin
FH active block at a basic and sidespin attack
BH active block at a basic topspin and sidespin attack
FH active block at a fast final (executed with max. force) topspin or smash
BH active block at a fast final (executed with max. force) topspin or smash
FH “drive” counterattack (attack without rotation) at a basic topspin or sidespin attack
BH “drive” counterattack (attack without rotation) at a basic topspin or sidespin attack
FH “drive” counterattack (attack without rotation) at a fast final (executed with max. force) topspin or a strong drive attack
BH “drive” counterattack (attack without rotation) at a fast final (executed with max. force) topspin or a strong drive attack
FH topspin counterattack at a basic topspin or sidespin attack
BH topspin counterattack at a basic topspin or sidespin attack
FH topspin counterattack at a fast final (executed with max. force) topspin
BH topspin counterattack at a fast final (executed with max. force) topspin
FH topspin counterattack at a fast final drive attack (attack without rotation)
BH topspin counterattack at a fast final drive attack (attack without rotation)
FH push “short at short”
BH push “short at short”
FH offensive chop at a chopped or on a pushed (ball without rotation) ball
BH offensive chop at a chopped or on a pushed (ball without rotation) ball
FH defensive chop at a chopped, pushed or backspin defense ball
BH defensive chop at a chopped, pushed or backspin defense ball
FH short ball at a backspin defense
BH short ball at a backspin defense
FH straight flat (saving) ball on a lower pushed, passive blocked or flat ball
BH straight flat (saving) ball on a lower pushed, passive blocked or flat ball
FH balloon defense without rotation at a flat, chopped or pushed ball
BH balloon defense without rotation at a flat, chopped or pushed ball
FH short chopped or sideward chopped service
BH short chopped or sideward chopped service
FH short topspin (forward rotation – upwards) or topspin sideward service
BH short topspin (forward rotation – upwards) or topspin sideward service
FH short “empty” (without rotation-plunged) service
BH short “empty” (without rotation-plunged) service
FH half long (service on baseline of the table) chopped (backspin rotation-downwards) or chopped sideward service
BH half long (service on base line of the table) chopped (backspin rotation-downwards) or chopped sideward service
FH half long topspin (forward rotation – upwards) or topspin sideward service
BH half long topspin (forward rotation – upwards) or topspin sideward service
FH half long “empty” flat (without rotation) service
BH half long “empty” flat (without rotation) service
FH long chopped backspin or chopped sideward service
BH long chopped backspin or chopped sideward service
FH long topspin (forward rotation-upwards) or sideward topspin service
BH long topspin (forward rotation-upwards) or sideward topspin service
FH long “empty” (without rotation-pushed) service
BH long “empty” (without rotation-pushed) service
FH return of short service short (chopped or pushed ball)
BH return of short service short (chopped or pushed ball)
FH return of short service with a long chop (chopped or pushed ball)
BH return of short service with a long chop (chopped or pushed ball)
FH attack over the table (“flick”) on a short service
BH attack over the table (“flick”) on a short service
FH drive attack at a half long service
FH topspin attack at a half long service
BH topspin attack at a half long service
FH return of a half long service short (chopped or pushed ball)
BH return of a half long service short (chopped or pushed ball)
FH return of a half long service with a chopped or pushed ball
BH return of a half long service with a chopped or pushed ball
FH drive attack at a long service
BH drive attack at a long service
FH topspin attack at a long service
BH topspin attack at a long service
FH return of a long service with a chopped or pushed ball
BH return of a long service with a chopped or pushed ball
